# Assessment of Knowledge, Attitude, Practice and Associated Factors Towards Sedation Among Nurses Working in Intensive Care Units of Comprehensive Specialized Hospitals: A Multicentre Cross‐Sectional Study

**DOI:** 10.1155/nrp/6421067

**Published:** 2026-05-26

**Authors:** Habtu Adane Aytolgn, Gashaye Kindalem Mersha, Nigussie Simeneh Endalew, Shimelis Seid Tegegne, Abatneh Feleke Agegnehu

**Affiliations:** ^1^ Department of Anesthesia and Critical Care, College of Medicine and Health Science, University of Gondar, Gondar, Ethiopia, uog.edu.et; ^2^ Department of Anesthesia, College of Medicine and Health Science, Debre Berhan University, Debre Birhan, Ethiopia, dbu.edu.et; ^3^ Department of Anesthesia, College of Health Science, Debre Tabor University, Debre Tabor, Ethiopia, dtu.edu.et

**Keywords:** attitude, ICU, knowledge, nurses, practice, sedation

## Abstract

**Background:**

Sedation is an important component of intensive care unit management. It is a sleep‐like state where patients are generally unaware of surroundings. The levels of knowledge, attitude and practice regarding sedation in the intensive care unit were not studied as a multicentre study in the Amhara National Regional State.

**Methods:**

A hospital‐based multicentre cross‐sectional study was conducted. The data were collected using survey with a self‐administered structured close‐ended questionnaire. Binary logistic regression analyses were used to identify factors associated with the level of knowledge, attitude and practice of nurses towards sedation.

**Result:**

The overall proportions of good levels of knowledge towards sedation in the ICU among nurses were 60.4% (CI = 53.8%–67%). The overall proportions of good levels of attitude towards sedation in the ICU among nurses were 69.8% (CI = 63.4%–75.6%). Furthermore, the proportions of adequate practice were 74.5% (CI = 68.5–80.3). Having a master’s degree (AOR = 8.9, CI = 3.5–22.7), receiving training towards sedation (AOR = 5.4, CI = 1.5–19.5), having a written protocol (AOR = 6.7, CI = 2.18–20.13) and having critical care certification (AOR = 9.9, CI = 2.93–46.92), respectively, were associated with a higher level of knowledge. Similarly, having a master’s degree (AOR = 4.3, CI = 2.2–12.4), receiving training towards sedation (AOR = 4.4, CI = 2.1–14.3) and having critical care certification (AOR = 6.6, CI = 2.5–10.7) were associated with a higher level of attitude. Receiving training towards sedation (AOR = 8.3, CI = 4.7–17.2), total work experience > 7 years and ICU work experience > 7 years were associated with a higher level of practice.

**Conclusion:**

This study emphasizes that increased sedation‐related knowledge, attitude and practice among ICU nurses are largely driven by institutional and professional variables, especially training, the availability of sedation protocols and having critical care certification. The results highlight the necessity of bolstering professional certification, protocol‐based care and structured training programmes to enhance the uniformity and calibre of sedation management in intensive care units. It is probably more effective to address these issues at the system level than to concentrate only on individual expertise.

## 1. Introduction

The intensive care unit (ICU) is a critical setting where patients with life‐threatening conditions require specialized care and support [1]. Sedation is one of the most important components of ICU management. Sedation is a sleep‐like state where patients are unaware of surroundings or environment in general but may still respond to external stimuli. It helps to alleviate patient discomfort, reduce the stress response, provide anxiolytics, improve tolerance of ventilator support, promote relaxation and facilitate nursing care in the ICU [2, 3].

Globally, around 90% of critical patients need mechanical ventilation and sedation during their stay in the ICU [4]. The indications for sedation and analgesia are individualized for each patient based on their condition in the ICU. There is no ideal sedative agent for sedation, so it may need different drugs to achieve comfort and to produce tolerance during nursing care [5–7].

Even though sedation is an integral part of critical care practice, if not given appropriately, it may have the potential to prolong mechanical ventilation, which in turn increases hospital stay and healthcare costs, and it may cause haemodynamic instability [3]. Sedation was focused on extensive use of sedative agents to achieve deep sedation or detachment from the environment. However, recent studies suggest that patient outcomes are significantly influenced by the choice of agent and the presence of over‐ and undersedation [8]. Therefore, it requires adequate knowledge, attitude and evidence‐based practice for healthcare workers [9].

Because of the high load of patient care, social isolation, risk of contamination and worry about patients’ futures, the ICU is the most stressful and anxiety‐inducing place for medical workers. This results in low self‐esteem and subpar work output, which causes unanticipated mistakes [10, 11]. As a result, consistent monitoring and procedure adherence are influenced by the working conditions [1]. Promising substitutes for conventional techniques, such as the Glasgow Coma Scale (GCS) and bispectral index, include various sedation scales, including the Riker Sedation‐Agitation Scale, Motor Activity Assessment Scale, Richmond Agitation‐Sedation Scale, and Ramsay Sedation Scale [12–14].

The ICU nurses usually work in the ICU for an extended period of time and are responsible for sedating and following critically ill patients [5]. However, studies conducted in different areas showed gap regarding adequate knowledge, attitude and practice [15, 16]. As a result, it is important to identify the level of gaps in order to improve the quality of management [5, 16] and to decrease the complications associated with sedation [17].

Thus, this study was aimed to assess the level of knowledge, attitude, practice and associated factors towards sedation among nurses working in the ICUs of comprehensive and specialized hospitals in the Amhara National Regional State, Ethiopia.

## 2. Method and Material

### 2.1. Study Design and Period

An institutional‐based multicentre cross‐sectional study was conducted from 16 July 2023 to 01 August 2023.

### 2.2. Study Area

The study was conducted at eight comprehensive and specialized hospitals in the Amhara National Regional State, Ethiopia. Those were the University of Gondar, Tibebe Ghion, Felege Hiwot, Debre Tabor, Debre Markos, Woldia, Dessie and Debre Berhan. In the Amhara National Regional Health Bureau’s Annual Performance Report, the region has 81 hospitals and 858 health centres: the University of Gondar (750 km northwest of Addis Ababa [the capital city of Ethiopia] having 26 ICU beds), Tibebe Ghion and Felege Hiwot (which are found in the capital city of the Amhara Region and 565 km northwest of the capital city having 30 ICU beds), Debre Tabor (665 km northwest of the capital city with 10 ICU beds), Debre Markos (296 km from the capital city with 3 ICU beds), Woldia and Dessie (520 km and 401 km, respectively, from the capital city of Ethiopia having 12 total ICU beds) and Debre Berhan (125 km from the capital city having 6 ICU beds) [18].

### 2.3. Source and Study Population

#### 2.3.1. Source Population

All nurses working at comprehensive and specialized hospitals were the source population, while the study population were nurses who were working in the ICU of comprehensive and specialized hospitals of the Amhara National Regional State.

#### 2.3.2. Eligibility Criteria

This study excluded nurses who worked in newborn intensive care units (NICUs) but included adult and paediatric ICU nurses. As both adult and paediatric ICU nurses manage sedation using identical clinical principles and protocols, data from both groups were evaluated together. Potential variations between these settings, however, were taken into account and are recognized as a restriction.

### 2.4. Study Variables

#### 2.4.1. Dependent Variables

Dependent variables are knowledge, attitude and practice of ICU nurses.

#### 2.4.2. Independent Variables

Sociodemographic factors: age, sex, work experience in the unit, educational status, total work experience and certification in critical care.

Institutional factors: availability of guidelines, availability of protocols and training on sedation protocols.

### 2.5. Operational Definitions

Knowledge: Good, if the total score of the knowledge response is above the mean value, and poor, if the score is below the mean value, based on the previous literature [15].

Attitude: Good, if the total score of attitude response is above the mean value, and poor, if the total score of attitude response is below the mean value, based on the previous literature [15].

Practice: Adequate, if the total score of the practice response is above the mean value, and inadequate, if the total score of the practice response is below the mean value, based on the previous literature [15].

The sum of the item responses was used to calculate the knowledge, attitude and practice scores. The mean score was utilized as a threshold to classify participants into good/poor knowledge and attitude and adequate/inadequate practice because there were no generally verified cutoff points for these categories in comparable circumstances. Although this method has been employed in similar studies, it is recognized as a weakness because it may decrease comparability and introduce misclassification.

### 2.6. Sample Technique

First, a sample size was determined using conventional statistical techniques. Nevertheless, the computed sample size was exceeded by the total number of eligible ICU nurses in the chosen institutions. As a result, a census method was used, and the study included all qualified ICU nurses. No sampling technique or sampling‐bias adjustment was used because every participant who was available was included.

### 2.7. Data Collection Procedures

Data were collected with an English version of a self‐administered questionnaire. Semi‐structured questionnaires consisting of knowledge, attitude and practice were adapted from different literature reviews [15, 19–23] and the theory of planned behaviour [24]. Practice was assessed using self‐reported responses, which may be subject to recall and social desirability bias. Six critical care experts determined the content validity and reliability of the knowledge questionnaire, and the calculated content validity index of the questionnaire was 0.90 [16]. The content validity of the practice and attitude questionnaire was (Cronbach’s a of) 0.8 [21]. The questionnaire consisted of majorly 39 questions with Cronbach’s alpha value of 0.89. Thus, demographic characteristics of nurses [6], training and institutional characteristics [6] and knowledge part had 12 questions with Cronbach’s alpha of 0.67. Thus, general knowledge of nurses towards sedation [9], knowledge of nurses towards over‐ and undersedation (3 with subquestions from A to E) and attitude of nurses towards sedation [7] were with Cronbach’s alpha of 0.86 and practice of nurses [9] with Cronbach’s alpha of 0.74.

### 2.8. Data Quality Control

Data were collected by trained data collectors, guided by supervisors. Training and orientation were given to data collectors and supervisors for one day with the aim of the study: how to approach study participants, how to use questionnaires, how to supervise and how to collect data. Pretasting was done with 5% of the sample with no significant modification before data collection at UOGCSH surgical ICU. The collected data were checked for completeness, accuracy and clarity.

### 2.9. Data Processing and Analysis Procedures

The data were coded, entered into EpiData Version 4.6 and exported to SPSS Version 25 for analysis; descriptive statistics were displayed using text, tables and graphs; bivariable logistic regression analysis was used to find candidate variables (*p* < 0.2) for multivariable logistic regression; multivariable binary logistic regression was then used to find factors independently associated with knowledge, attitude and practice; statistical significance was declared at *p* < 0.05 with 95% confidence intervals; the Hosmer–Lemeshow goodness‐of‐fit test; multicollinearity was examined using variance inflation factor (VIF), with a threshold of VIF < 10 was deemed acceptable; and missing data were handled using complete case analysis.

## 3. Results

After checking the reliability (Cronbach’s alpha) of separate questions of knowledge, attitude and practice, the obtained values were 0.67, 0.86 and 0.764, respectively. The overall Cronbach’s alpha was 0.89.

### 3.1. Sociodemographic Characteristics

In this study, 212 nurses were accessible and participated during the data collection with a response rate of 93.4%. Of the total, 104 were males, and 108 were females (Table 1).

**TABLE 1:** Sociodemographic characteristics of nurses working in the ICU at comprehensive and specialized hospitals in the Amhara National Regional State, Ethiopia, 2023 (*N* = 212).

Variables	Frequency	Percentage (%)
Age (years):		
20–30	89	40
31–40	83	39
> 40	40	19
Sex:		
Male	104	49
Female	108	51
Level of education:		
Diploma nurse	32	15
Bachelor degree	113	53
Postbasic degree in ICU	20	9
Master’s degree and above	47	22
ICU work experience:		
< 2	118	57
2–4	36	17
4–7	37	17
> 7	21	10
Total work experience:		
< 2	28	13
2–4	32	15
4–7	52	24
> 7	100	47

Abbreviation: ICU, intensive care unit.

### 3.2. Work‐ and ICU‐Related Characteristics

In this study, only 27.8% of respondents had critical care certification, and only 22.2% of nurses had training regarding sedation in the ICU. Only 26.4% of nurses used sedation assessment tool during sedation practice. Of those, most of the respondents used the GCS score (37.5%) to assess the level of sedation (Table 2).

**TABLE 2:** Work‐ and ICU‐related characteristics of ICU nurses in comprehensive and specialized hospitals in the Amhara National Regional State, Ethiopia (*N* = 212).

Comprehensive and specialized hospitals	Frequency (*n*)	Percentage (%)
UOGCSH	31	14.6
TGCSH	29	13.7
FHCSH	25	11.8
DTCSH	29	13.7
DMCSH	22	10.4
WCSH	23	10.8
DCSH	25	11.8
DBCSH	28	13

### 3.3. Institutional‐Related Characteristics

Most of the nurses working in the ICU had no training or critical care certificate. Furthermore, 74.1% of them did not use sedation protocols. The GCS score was utilized by the majority of responders (37.5%) to gauge the degree of sedation (see Table 3). The GCS is not a validated sedative assessment instrument; rather, it is largely a neurological examination tool. This result raises the possibility that ICU staff are ignorant of or misinformed about the proper sedation monitoring instruments.

**TABLE 3:** Institutional‐related characteristics of ICU nurses working in comprehensive and specialized hospitals in the Amhara National Regional State, Ethiopia (*N* = 212).

Variables	Frequency (*n*)	Percentage (%)
Critical care certification:		
Yes	59	27.8
No	153	72.2
Training regarding sedation:		
Yes	47	22.2
No	165	78.8
Availability of sedation protocol Or and guidelines:		
Yes	55	25.9
No	157	74.1
Sedation assessment tool used:		
Glasgow Coma Scale score	21	37.5
Riker Sedation‐Agitation Scale score	11	19.6
Richmond Sedation‐Agitation Scale score	8	14.3
Ramsey score	16	28.6
Total	56	100.0

### 3.4. Knowledge of Nurses Towards Sedation in the ICU

Of the total, 84 (39.6%) (CI = 33.0–46.2) of respondents had poor knowledge, and 128 (60.4) (CI = 53.8–67) had good knowledge towards sedation and its management in the ICU.

The most correctly answered questions were oversedation causing respiratory depression (*n* = 199, 93.9%) followed by undersedation causing tachypnoea (*n* = 182, 85.8%), while the least answered questions were undersedated individuals moving their trunk or lifting their legs off the bed (*n* = 69, 32.5%), followed by opioids and benzodiazepines having a potential to cause withdrawal effects after the use of approximately seven days (*n* = 78, 36.8%). The identification of indicators of undersedation and the effects of drug withdrawal had the lowest percentage of properly answered questions. These results can point to deficiencies in focused training as well as a lack of access to the application of standardized sedation procedures in clinical settings.

### 3.5. Factors Associated With Knowledge Level of Nurses Towards Sedation

In the bivariable logistic regression model, age, level of education, received training about sedation, availability of a written sedation protocol or guidelines, having critical care certification, having good attitude, having adequate level of practice, total work experience and ICU work experience were found to be statistically significant, *p* value < 0.2, and were fitted to multiple binary logistic regressions. At the end of multiple binary logistic regressions, level of education, received training, sedation protocols, having critical care certification, attitude level and total work experience were significantly associated with the level of knowledge.

The likelihood of having a higher level of knowledge among nurses towards sedation with a master’s degree was 8.9 times higher than those who had a diploma (AOR = 8.9, CI = 2.2–12.4). Moreover, the odds of having a higher level of knowledge among nurses receiving training towards sedation were 5.4 times higher than those who did not receive training (AOR = 5.4, 1.5–19.5). This shows that training is vital to improve ICU sedation.

Furthermore, the likelihood of having a higher level of knowledge among nurses who had a written sedation protocol in the clinical area was 6.7 times higher than those who did not have a written sedation protocol (AOR = 6.7, 2.2–20.1).

In addition, the odds of having a higher level of knowledge among nurses who have critical care certification were 9.9 (AOR = 9.9, 2.9–46.9) times more than those who did not have critical care certification. In the same way, the odds of having a higher level of knowledge among nurses who had a good level of attitude were 3.5 times higher than those who had poor level of attitude (AOR = 3.5,1.2–10.6). The nurses who also had greater than 7 years of work experience had more than 12 times the likelihood of having good knowledge than those with less than 7 years (Table 4).

**TABLE 4:** Binary logistic regression for knowledge levels of nurses towards sedation working in the ICU in comprehensive and specialized hospitals in the Amhara National Regional State, Ethiopia, 2023 (*N* = 212).

Variables	Level of knowledge		COR (95% CI)	AOR (95% CI)	*p* value
	Good (%)	Poor (%)			
Age (years)					
> 40	28	12	9.44 (6.35–23.31)	2.06 (0.24–17.89)	0.51
30–40	54	29	2.74 (1.48–5.09)	2.16 (0.09–1.31)	0.12
20–30	36	53	1	1	
Level of education:					
Master’s degree and above	41	6	11.38 (10.99–34.38)	8.98 (1.39–57.87)	0.021^∗^
Postbasic degree in ICU	12	8	2.5 (1.62–9.47)	1.21 (0.132–4.08)	0.78
Bachelor degree	64	49	2.17 (1.36–14.94)	1.42 (0.32–4.54)	0.72
Diploma nurse	12	20	1	1	
Training towards sedation:					
Yes	41	6	6.13 (2.47–15.21)	5.41 (1.5–19.51)	0.010^∗^
No	87	78	1	1	
Availability of sedation protocol:					
Yes	42	13	2.66 (1.33–5.36)	6.66 (2.18–20.13)	< 0.001^∗∗^
No	86	71	1	1	
Having critical care certification:					
Yes	53	6	9.19 (3.73–22.64)	9.93 (2.93–46.92)	< 0.001^∗∗^
No	75	78	1	1	
Attitude towards sedation:					
Good	122	26	6.86 (2.03–8.31)	3.53 (1.18–10.61)	0.025^∗^
Poor	26	38	1	1	
Level of practice:					
Adequate	106	52	3.20 (1.44–5.98)	1.94 (0.62–6.11)	0.076
Inadequate	21	33	1	1	
Total work experience (years):					
> 7	74	26	10.44 (8.38–53.45)	12.56 (4.12–46.63)	0.002^∗^
4–7	31	21	5.41 (4.11–36.23)	8.51 (2.49–28.9)	0.004^∗^
2–4	13	19	2.51 (2.32–25.64)	4.63 (0.92–23.6)	0.06
< 2	6	22	1	1	
ICU experience (years):					
> 7	17	4	5.39 (2.03–26.25)	2.77 (0.57–13.38)	0.20
4–7	25	12	2.64 (3.54–31.90)	1.45 (0.20–10.70)	0.70
2–4	23	13	2.24 (1.48–7.56)	1.19 (0.32–4.49)	0.79
< 2	52	66	1	1	

^∗^ *p* < 0.05.

^∗∗^ *p* < 0.001.

### 3.6. The Attitude Level of Nurses Towards Sedation

Of the study participants, 148 (69.8) (CI = 63.4–75.6) had good attitude towards sedation, and 64 (30.2) (CI = 24.1–36.3) had poor attitude towards sedation. In response to attitude questions, the majority of participants strongly agreed that mechanical ventilation is uncomfortable and stressful to the patient (*n* = 179, 84.4%).

### 3.7. Factors Associated With Attitude Level of Nurses Towards Sedation

In the bivariable logistic regression analysis, level of education, training towards sedation, availability of written protocol, having critical care certification, ICU work experience, total work experience, good level of knowledge and adequate practice were found to be statistically significant with *p* value < 0.2. Finally, receiving training towards sedation and having critical care certification and education level were associated with good attitude in multiple logistic regressions.

Thus, nurses with a master’s degree were 4.3 times more likely to have good attitude than those with a diploma (AOR = 4.3, CI = 2.2–12.4). Similarly, nurses who received training about sedation were 4.4 times more likely to have good attitude than those who did not receive training (AOR = 4.4, 2.1–14.3). Furthermore, the nurses who had critical care certification were 6.6 times more likely to have good attitude than those who had not (AOR = 6.6, 2.5–10.7) (see Table 5).

**TABLE 5:** Binary logistic regression for attitude levels of nurses towards sedation working in the ICU of comprehensive and specialized hospitals in the Amhara National Regional State, Ethiopia, 2023 (*N* = 212).

Variables	Attitude level		COR 95% CR	AOR 95% CR	*p* value
	Good (%)	Poor (%)			
Training towards sedation:					
Yes	39	8	4.93 (1.4–8.9)	4.44 (2.1–14.3)	0.001^∗^
No	82	83	1	1	
Having critical care certification:					
Yes	51	8	6.99 (3.12–8.81)	6.57 (2.5–10.72)	0.002^∗^
No	73	80	1	1	
Level of education:					
Master’s degree and above	38	9	5.43 (4.12–21.56)	4.32 (2.2–12.4)	0.003^∗^
Postbasic degree	12	8	1.93 (0.97–3.36)	1.42 (0.87–13.43)	0.098
Bachelor degree	66	47	1.81 (0.67–4.73)	1.21 (0.64–5.34)	0.289
Diploma nurse	14	18	1	1	

^∗^ *p* < 0.05.

### 3.8. Self‐Reported Practice Level of Nurses Towards Sedation

Based on self‐reported responses, 158 (74.5%) of respondents had adequate practice. About 94 (44.3%) of respondents consistently monitor oxygen saturation before and during sedation followed by 86 (40.6%) of respondents monitoring respiratory rate before and during sedation.

### 3.9. Factors Associated With Practice Level of Nurses Towards Sedation

In the bivariable logistic regression analysis, level of education, training towards sedation, having critical care certification, availability of written protocol, ICU work experience, total work experience and good level of knowledge were found to be statistically significant, *p* value < 0.2. As a result, received training, total work experience and ICU work experience were associated with a higher level of practice in multiple binary logistic regressions.

Accordingly, the likelihood of having a higher level of practice among nurses who had received training related to sedation was 8.3 times higher than those who had not received training (AOR = 8.3, 4.7–17.2).

In addition, the likelihood of having a higher level of practice among nurses who have > 7 years of total work experience and ICU work experience was 4.3 (AOR = 4.3,1.6–10.9) and 4.2 (AOR = 4.2,1.1–6.3) times higher than those who have less than 2 years of experience, respectively (see Table 6).

**TABLE 6:** Binary logistic regression for practice levels of nurses towards sedation in the ICU of comprehensive and specialized hospitals in the Amhara National Regional State, Ethiopia, 2023 (*N* = 212).

Variables	Level of practice		COR (95% CI)	AOR (95% CI)	*p* value
	Adequate	Inadequate			
Training towards sedation					
Yes	42	5	8.71 (3.5–12.6)	8.32 (4.7–17.2)	< 0.001^∗∗^
No	81	84	1	1	
Total work experience (in years)					
> 7	72	28	5.43 (4.8–11.4)	4.3 (1.6–10.9)	0.02^∗^
4–7	29	23	2.66 (1.4–7.9)	2.3 (0.8–6.4)	0.089
2–4	15	17	1.86 (1.2–9.6)	1.3 (0.7–1.7)	0.434
< 2	9	19	1	1	
ICU work experience (in years)					
> 7	16	5	4.66 (2.9–8.8)	4.21 (1.1–6.3)	0.03^∗^
4–7	23	15	2.24 (1.3–6.7)	2.13 (0.6–7.2)	0.105
2–4	20	16	1.83 (1.1–5.4)	1.21 (0.1–13.1	0.409
< 2	48	70	1	1	

^∗^ *p* < 0.05.

^∗∗^ *p* < 0.001.

## 4. Discussion

In this study, the overall proportions of poor and good levels of knowledge towards sedation in the ICU were 39.6% and 60.4%, respectively. This is comparable to the study conducted in Pakistan, which showed that 33% and 67% had poor and good knowledge towards sedation, respectively [16]. In this study, knowledge level was lower than the study conducted in Canada which reported that 85.3% of respondents had good levels of knowledge in the management of sedation in the ICU [25]. It is also slightly lower than the study conducted in Kenya National Hospital which reported that 75.9% of respondents had good level of knowledge [15].

In our study, only 26.4% of nurses employed sedation evaluation tools, and the majority of them relied on the GCS, which is not a validated sedation scale, despite the fact that 60.4% of nurses had good understanding, and 74.5% indicated adequate practice. This implies that knowledge does not always result in evidence‐based practice.

In this recent study, having a master’s degree has a higher association with a good level of knowledge than having a diploma in nursing. This finding was in line with the studies conducted in Australia and New Zealand [26, 27]. This is also supported by the study conducted in Boston [28]. The similarities might be due to increased capacity for reading, the education curriculum and the study area being the university hospital.

In this study, good level of knowledge and receiving training towards sedation had strong association. This is in line with the study conducted in Denmark, which showed that those who received training on sedation had a higher level of knowledge than those who did not receive training [29]. This is also supported by the study in Tanzania [30], while studies conducted in the United States and Canada were against the recent study [25, 28]. The discrepancy may be due to their exposure to clinical practice becoming longer, which might allow them to communicate with their colleagues, share information and acquire knowledge regarding sedation. These results are in line with those of Bardhia et al. (2025), who showed that patient‐centred care in ICUs is greatly enhanced by a supportive nurse practice environment that includes institutional support, enough staffing and leadership. This is consistent with the current investigation, which found that inadequate training and a lack of protocols were associated with less‐than‐ideal sedation techniques.

In this study, having a written protocol in their clinical area had a strong association with having good knowledge. This is in line with the study conducted in the United Kingdom [3]. This is also supported by the study conducted in Kenya [15]. This similarity might be that the availability of protocol in their work environment might contain recent and up‐to‐date findings that are sources of knowledge and better patient care. System‐level limitations, such as heavy workloads, understaffing, a lack of protocols and unstructured monitoring systems, most likely account for this disparity. According to Daraghmeh et al. (2024), missed nursing care in ICUs was significantly correlated with workload and resource constraints, which had an impact on the delivery of standard care.

In this study, having critical care certification and having good attitude had a strong relationship with a good level of knowledge; this is supported by the study conducted in the United States [28, 31]. This similarity may be due to acquiring knowledge during critical care certification.

In this recent study, total work experience was strongly associated with the level of knowledge. This is supported by other studies conducted in the United States [28], Canada [25] and Denmark [29]. This similarity may be due to work experience being a source of knowledge.

In this study, the overall proportions of poor and good levels of attitude towards sedation in the ICU were 30.2% and 69.8%, respectively. This is also comparable with a study conducted in the United States among critical care nurses that showed 66% of respondents had good attitude, and 34% of respondents had poor attitude towards sedation [21].

In this study, the overall attitude towards sedation, the level of education having a master’s degree, training towards sedation and having critical care certification were statistically significant (*p* value < 0.05). This is comparable with the study conducted in the United Kingdom [3].

In this study, the overall proportions of inadequate and adequate levels of practice towards sedation in the ICU were 25.5% and 74.5%, respectively. This is also comparable with a study conducted in Egypt, which reported that 64% of the nurses had satisfactory practice, while 34% of the nurses under study had unsatisfactory practice levels [32]. This similarity may be due to the fact that both of the studies were conducted in the university hospitals.

In this study, the overall practice towards sedation was strongly associated with training towards sedation, total work experience and ICU work experience. This is supported by studies conducted in Denmark [33] and Australia [34]. This similarity may be due to total work and ICU experience increasing the practice level of nurses by increasing their exposure to different critically ill patients.

## 5. Conclusion

This study emphasizes that increased sedation‐related knowledge, attitude and practice among ICU nurses are largely driven by institutional and professional variables, especially training, the availability of sedation protocols and having critical care certification. The results highlight the necessity of bolstering professional certification, protocol‐based care and structured training programmes to enhance the uniformity and calibre of sedation management in ICUs. It is probably more effective to address these issues at the system level than to concentrate only on individual expertise.

### 5.1. Limitations of the Study

Due to the differences in approaches to adult and paediatric patient care in ICU sedation, this study does not specify the clinician’s levels of knowledge, attitude and practice on these specific population groups. The other limitations may be self‐reported practice as a potential source of bias. The other limitations might be potential social desirability bias, mixing adult/paediatric settings as a confounder, measurement reliability issues and low sample size.

## 6. Recommendations

There is still an unsatisfactory level of knowledge, attitude and practice among nurses. Therefore, focusing on the preparation of updates, special training and adopting and preparing local guidelines and protocols is recommended to improve the knowledge and practice level of ICU nurses towards sedation. Therefore, we recommend that policymakers prepare regular training involving the latest updates of sedation guidelines. For hospital administrators, we suggest preparing ICU sedation protocols, organizing regular training and assigning more experienced nurses to the ICU. Further research incorporating a large sample size, observing clinical practice, adherence with protocols and the effect of sedation practice on the outcome of patients’ needs to be done.

**Nomenclature**
ACCM: American College of Critical Care Medicine
DBCSH: Debre Berhan comprehensive and specialized hospital
DCSH: Dessie comprehensive and specialized hospital
DBTCSH: Debre Tabor comprehensive and specialized hospital
DMCSH: Debre Markos comprehensive and specialized hospital
FHCSH: Felege Hiwot comprehensive and specialized hospital
ICU: intensive care unit
SCCM: Society of Critical Care Medicine
TGCSH: Tibebe Gion comprehensive and specialized hospital
UGCSH: University of Gondar comprehensive and specialized hospital
UK: United Kingdom
USA: United States of America
WCSH: Woldia comprehensive and specialized hospital

## Author Contributions

Habtu Adane Aytolgn, Gashaye Kindalem Mersha, Nigussie Simeneh Endalew, Shimelis Seid Tegegne and Abatneh Feleke Agegnehu all contributed to the study’s idea and design, data collection, analysis and interpretation and manuscript drafting.

## Funding

This study was not supported by any public, private or nonprofit funding bodies.

## Disclosure

All authors gave final approval of the manuscript version.

## Ethics Statement

This study was conducted in accordance with the Declaration of Helsinki. Ethical clearance was obtained from the University of Gondar comprehensive and specialized hospital ethical review committee. The participants disclosed the purpose of the study, study procedures, and benefit before written informed consent was obtained. Each participant was informed that they have a full right to participate or decline from participating in the study. The participant’s confidentiality was ensured that all data collected were confidentially secured by avoiding their name and other personally identifying information.

## Conflicts of Interest

The authors declare no conflicts of interest.

## Data Availability

The original data are available in the hands of the corresponding author and will be shared based on your request.
